# Centenarians in the Global South: A View from Ethiopia

**DOI:** 10.1093/geroni/igab046.3027

**Published:** 2021-12-17

**Authors:** Samson Chane, Margaret Adamek

**Affiliations:** 1 Bahir Dar University, Bahir Dar, Oromiya, Ethiopia; 2 Indiana University, INDIANAPOLIS, Indiana, United States

## Abstract

As global aging advances, the number of centenarians worldwide is greatly increasing. Most of what is known about centenarians comes the Global North. It is not clear what factors contribute to longevity of centenarians in impoverished, mostly rural areas of Global South nations that still lack basic amenities. Cultural differences in the profile, lifestyles, and needs of centenarians in Africa have yet to be documented. Using a case study design, this descriptive inquiry investigated the profiles of centenarians in Ethiopia including religion, marriage, education, occupation, income, and living arrangement. Data were generated through in-depth interviews with nine centenarians (1 woman, 8 men) and were analyzed using descriptive narrative analysis. Respondents were between 100 and 108 years old. All nine were adherents of Orthodox Christianity, had been married, and were great-grandparents. Their adult lives were marked by both residential and marital stability. The Ethiopian centenarians persevered through many losses and hardships with the help of strong community-based social networks. Unlike studies of centenarians in the Global North, most respondents were male and had strict religious upbringings. Understanding the unique profiles of centenarians in the Global South will help to inform research and practice with this growing population of the oldest-old.

